# Census Bureau Household Panel

**DOI:** 10.23889/ijpds.v9i5.2916

**Published:** 2024-09-18

**Authors:** Jason Fields

**Affiliations:** 1 Census Bureau

The Census Bureau Household Panel is a nationally-representative, address-based, probability-based internet panel (including non-internet households) designed to improve representativeness, significantly reduce burden on sampled households, and promote high-frequency data meeting the immediate needs of the government and public. The Panel facilitates near real-time analysis of national events that may impact social, economic, or demographic characteristics of the population. It supports research to improve surveys, including testing content changes, alternative methods for enhancing data with administrative and other data sources, and adaptive survey design procedures.

